# Nonlinear Association of Glycosylated Hemoglobin With Single Intracranial Aneurysm Rupture in Patients With Diabetes Mellitus: A Cross-Sectional Study

**DOI:** 10.3389/fneur.2022.854008

**Published:** 2022-03-28

**Authors:** Shi-Xing Su, Xue-Tao Wang, Xi-Feng Li, Chuan-Zhi Duan, Yi-Ming Bi, Xin Zhang

**Affiliations:** ^1^National Key Clinical Specialty/Engineering Technology Research Center of Education Ministry of China, Guangdong Provincial Key Laboratory on Brain Function Repair and Regeneration, Department of Neurosurgery, Neurosurgery Institute, Zhujiang Hospital, Southern Medical University, Guangzhou, China; ^2^Department of Neurosurgery, Zhongshan Hospital of Traditional Chinese Medicine, Zhongshan, China; ^3^Department of Interventional Treatment, Southern Medical University, Guangzhou, China

**Keywords:** hemoglobin A1c (HbA1c), association, single intracranial aneurysm, nonlinearity, diabetes mellitus

## Abstract

**Background:**

The published literature linking diabetes mellitus (DM) to intracranial aneurysm (IA) ruptured has been controversial and limited by methodology. Thus, this study was performed to examine whether hyperglycemia control status is independently associated with single IA rupture in patients with DM.

**Methods:**

We conducted a cross-sectional study on two Chinese hospitals between January 2010 and November 2017. Medical records of 223 patients with single IA and DM were reviewed and analyzed. We used glycosylated hemoglobin (GHB) as the independent variable of interest, and the outcome variable was ruptured status of IA. Covariates included data on demographics, morphological parameters, lifestyle habits, clinical features, and comorbidities.

**Results:**

Multivariable adjusted binary logistic regression and sensitivity analyses indicated that GHB was not associated with IA rupture (odds ratio OR, = 1.07, 95% CI 0.84–1.35). A nonlinear association between GHB and IA rupture was observed, whose inflection points were 5.5 and 8.9. The OR values (95% confidence intervals) were 0.38 (0.16–0.9) at the range of 1.88–5.5% of GHB, 1.6 (1.03, 2.5) at the range of 5.5–8.9%, and 0.56 (0.06–5.34) at the range of 8.9–10.1, respectively.

**Conclusion:**

The independent correlation between GHB and risk of IA rupture presented is nonlinear. The good glycemic control in single IA patients with DM can reduce the risk of IA rupture, and vice versa.

## Introduction

Previous animal studies have suggested that long-term hyperglycemia duration is associated with intracranial aneurysm (IA) pathogenesis (1–5). However, findings obtained from a review indicates that diabetes mellitus (DM) may be a protective factor for IA rupture (6). Can et al. (7) attributed these paradoxical results to methodological flaws. They suggest that lack of reliability of blood glucose level measurements in patients with ruptured IA is an important limitation (7).

Glycated hemoglobin (GHB, main HbA1c) is a reliable indicator for reflecting long-term blood glucose control (8). Glycated hemoglobin is more stable compared to fasting and random blood glucose. Given that published literature reporting DM mellitus duration can reduce the risk of IA rupture has been limited by methodology, using more reliable indicators to address this issue is needed.

We performed a large-scale cross-sectional study to investigate the association between GHB and rupture risk in Chinese patients with single IA.

## Participants and Methods

### Study Design

This is a cross-sectional study. We used GHB as the independent variable of interest to explore whether it was independently associated with the rupture status of single IA (dichotomous variable: 1 = rupture, 0 = unruptured).

### Study Population

A total of 1,847 patients with single IA were non-selectively and consecutively collected between January 2010 and November 2017 at the Department of Neurosurgery of Zhujiang Hospital, Southern Medical University, Guangzhou city, China and the Department of Neurosurgery, the First Affiliated Hospital of Zhengzhou University, Zhengzhou University, Zhengzhou City, China. Our inclusion criteria were: (1) patients diagnosed with IA by digital subtraction angiography (DSA); (2) patients with a clear history of diabetes (based on previous medical or health checkup records), and (3) patients taking/not taking blood sugar control drugs. Our exclusion criteria were as follows: (1) patients with multiple IAs, (2) patients with feeding artery aneurysm-associated arteriovenous malformations; (3) patients with fusiform IA; (4) patients with dissecting aneurysms; (5) patients with previous subarachnoid hemorrhage (SAH) history. After screening with the above inclusion and exclusion criteria, 223 patients with IA were left for data analysis (Supplementary Figure 1 for flowchart). The clinical information of the patients that were left were compiled from hospital electronic medical record systems. Informed consent of the participants was not required in this study because of the retrospective nature of the study. The hospital institutional review boards of the two institutions approved this study.

### Variables

We obtained baseline GHB and recorded it as continuous variable. The measurement of GHB was tested by the central laboratories of the hospitals. The detailed process of definition of IA rupture was described in our published reports (9).

The covariates used in this study can be classified as follows:

1. demographic data: age (year) and sex (male, female);
2. morphological factors (9, 10): position (PcoA: posterior communicating artery; AcoA: anterior communicating artery; ICA: internal carotid artery; ACA: anterior cerebral artery; MCA: middle cerebral artery; VA: vertebrobasilar artery), Willis variation (yes, no), shape of IA (regular or irregular), and neck status of IA (henceforth neck; wide or narrow);
3. other risk factors examined associated with rupture of IA (11–19): cerebral microbleed (identified by T2-weighted gradient-recalled-echo sequence on MRI, coded as yes or no), hypertension history (yes, no), DM history (yes, no), atherosclerosis (yes, no), and hyperlipidemia (yes, no);
4. risk factors according to our clinical experience: coronary artery disease (CAD, yes, no), current smoker (yes, no), current alcohol consumption (yes, no), and duration time of diabetes (year).

### Statistical Analysis

We presented continuous variables in two forms. In the first form, we expressed continuous variables with normal distribution as mean ± standard deviation. In the second form, we presented continuous variables with skewed distribution as medium (min, max). Categorical variables were expressed in frequency or percentage. We used χ^2^ (categorical variables), and performed Student *t*-test (normal distribution) or Mann-Whitney *U*-test (skewed distribution) to test for differences between the non-rupture and rupture IA groups.

Addressing linear relationship: univariate and multivariate binary logistic regressions were employed. We constructed two models: model 1 (no covariates were adjusted), and model 2 (covariates presented in Table 1 were adjusted).

**Table 1:** Baseline characteristics of patients with intracranial aneurysm (IA) and diabetes history.

***N***	**Unmatched cohort**		***P*-value**	**PSM cohort**		**Standardized**	***P*-value**
						**differences**	
	**Unruptured IA**	**Ruptured IA**		**Unruptured IA**	**Ruptured IA**		
	**139**	**84**		**64**	**64**		
**Demographics**
Age, mean ± sd, year	58.99 ± 11.25	58.15 ± 12.76	0.609	57.81 ± 12.47	59.38 ± 12.31	0.126	0.4770
Gender, No (%)			0.010			0.189	0.375
Male	81 (58.27%)	34 (40.48%)		38 (59.4%)	32 (50.00%)		
Female	58 (41.73%)	50 (59.52%)		26 (40.6%)	32 (50.00%)		
**Imaging parameters**
IA size, mean ± sd, mm	6.29 ± 3.68	6.33 ± 3.63	0.928	6.34 ± 3.82	6.27 ± 3.80	0.0176	0.9207
AR, mean ± sd	2.63 ± 0.81	2.30 ± 0.84	0.005	2.44 ± 0.82	2.48 ± 0.83	0.0569	0.748
IA Angle, mean ± sd, degree	131.39 ± 29.76	136.25 ± 32.13	0.253	132.09 ± 32.86	132.93 ± 33.79	0.025	0.886
SR, mean ± sd	2.74 ± 1.19	2.57 ± 0.95	0.263	2.56 ± 1.12	2.55 ± 0.99	0.0117	0.9472
Aneurysm location, No (%)			<0.001				0.0557
AcoA	26 (18.71%)	9 (10.71%)		11 (17.2%)	8 (12.5%)	0.1321	
ICA	28 (20.14%)	13 (15.48%)		9 (14.1%)	12 (18.8%)	0.1268	
ACA	10 (7.19%)	3 (3.57%)		3 (4.7%)	2 (3.1%)	0.0807	
VA	31 (22.30%)	3 (3.57%)		14 (21.9%)	3 (4.7%)	0.5235	
PcoA	37 (26.62%)	48 (57.14%)		22 (34.4%)	34 (53.1%)	0.3849	
MCA	7 (5.04%)	8 (9.52%)		5 (7.8%)	5 (7.8%)	0.0000	
CMBs, No (%)			0.176			0.0000	1.0000
No	131 (94.24%)	75 (89.29%)		58 (90.6%)	58 (90.6%)		
Yes	8 (5.76%)	9 (10.71%)		6 (9.4%)	6 (9.4%)		
Willis variation, No (%)			0.757			0.0383	1.0000
No	29 (20.86%)	19 (22.62%)		13 (20.3%)	14 (21.9%)		
Yes	110 (79.14%)	65 (77.38%)		51 (79.7%)	50 (78.1%)		
Aneurysm shape, No (%)			0.010			0.0658	0.8524
Regular	101 (72.66%)	47 (55.95%)		43 (67.2%)	41 (64.1%)		
Irregular	38 (27.34%)	37 (44.05%)		21 (32.8%)	23 (35.9%)		
Aneurysm neck, No (%)			<0.001			0.0335	1.0000
Wide	30 (21.58%)	37 (44.05%)		20 (31.2%)	21 (32.8%)		
Narrow	109 (78.42%)	47 (55.95%)		44 (68.8%)	43 (67.2%)		
**Target independent variable**
GHB, mean ±s d,%	7.18 ± 1.76	7.46 ± 1.71	0.250	–	–	–	–
**Cardiovascular risk factors**
Duration of diabetes	9.00 (1.00–30.00)	9.00 (1.00–26.00)	0.678	8.00 (1.00–26.00)	8.00 (1.00–28.00	0.1387	0.4343
Atherosclerosis, No (%)			0.069			0.0422	1.0000
No	107 (76.98%)	73 (86.90%)		53 (82.8%)	54 (84.4%)		
Yes	32 (23.02%)	11 (13.10%)		11 (17.2%)	10 (15.6%)		
Hyperlipidemia, No (%)			0.420			0.0324	1.0000
No	87 (62.59%)	48 (57.14%)		40 (62.5%)	41 (64.1%)		
Yes	52 (37.41%)	36 (42.86%)		24 (37.5%)	23 (35.9%)		
CAD, No (%)			<0.001			0.1268	0.6331
No	126 (90.65%)	59 (70.24%)		52 (81.2%)	55 (85.9%)		
Yes	13 (9.35%)	25 (29.76%)		12 (18.8%)	9 (14.1%)		
Current smoker, No (%)			0.086			0.1073	0.6861
No	102 (73.38%)	70 (83.33%)		49 (76.6%)	46 (71.9%)		
Yes	37 (26.62%)	14 (16.67%)		15 (23.4%)	18 (28.1%)		
Current drinker, No (%)			0.098			0.0383	1.0000
No	114 (82.01%)	61 (72.62%)		51 (79.7%)	50 (78.1%)		
Yes	25 (17.99%)	23 (27.38%)		13 (20.3%)	14 (21.9%)		
Hypertension history, No (%)			0.340			0.0633	0.8580
No	77 (55.40%)	41 (48.81%)		36 (56.2%)	38 (59.4%)		
Yes	62 (44.60%)	43 (51.19%)		28 (43.8%)	26 (40.6%)		

*CMB, cerebral microbleed; AR, aspect ratio; SR, size ratio; PcoA, posterior communicating artery; AcoA, anterior communicating artery; ICA, internal carotid artery; ACA, anterior cerebral artery; MCA, middle cerebral artery; VA, vertebrobasilar artery; CAD, coronary artery disease; GHB, glycosylated hemoglobin (HbA1c)*.

Addressing nonlinearity: to address nonlinearity of GHB and rupture risk, a generalized additive model was used, and smooth curve fitting (penalized spline method) was conducted. If nonlinearity was detected, we calculated the inflection point first using a recursive algorithm and then constructed a two-piecewise binary logistic regression model on both sides of the inflection point. In the end, the model that was more suitable for fitting (standard binary logistic regression model vs. two-piecewise model) the association between the target independent variable and the outcome variable was mainly determined by log likelihood ratio test.

### Sensitivity Analysis

To ensure the robustness of the data analysis, we did a series of sensitivity analyses including:

1. we converted GHB into a categorical variable according to quartile, and calculated the P for trend. The purpose was to verify the results of GHB as the continuous variable and to observe the possibility of nonlinearity.
2. Due to differences in baseline characteristics, propensity score (PS) matching was performed to ensure that patients with ruptured IA and those with unruptured IA had similar baseline characteristics (20, 21). PS was calculated with a multivariable logistic regression model. The parameters, which were used to estimate PS, are listed in Supplementary Table 1. A balanced evaluation of post-PS matching is shown in Table 1.
3. We considered that some eligible patients were unmatched during the matching process. Therefore, to prevent selection bias caused by patient lost, additional exploratory analyses by inverse-probability treatment weighting (IPTW) based on PS were performed. The equation of weight was as follows: when patients have no ruptured IA, weight = 1/(1–PS), and when patients have a ruptured IA, weight = 1/PS. Weighted binary logistic regression models were, therefore, used to estimate odds ratio (OR) and 95% confidential interval (CI) (22).

It was noted that the purpose of both PS matching and IPTW using was only to verify the results of multivariate logistic regression (the matching variable is IA rupture), rather than in real-world research which were used for the purpose of post hoc randomization (the matching variable is exposure variable) (20).

All the analyses were performed with the statistical software package R (http://www.R-project.org, The R Foundation). *P*-values < 0.05 (two-sided) were considered statistically significant.

## Results

### Baseline Characteristics of Selected Participants

In this study, a total of 84 cases were ruptured IA (84/223, 37.67%). Table 1 describes the baseline characteristics of the study population across categories of IA with or without rupture at admission. The average age of the 223 participants was 58.68 ± 11.8 years, and about 51.57% of the participants were male. No statistically significant differences were detected in age, IA size, size ratio, GHB, CMBs, atherosclerosis, hyperlipidemia, current smoking status, current alcoholic use, Willis variation, and hypertension history between patients with DM and IA rupture and those with DM but without IA rupture (all *P*-values > 0.05). Compared to non-rupture group, participants in rupture group had lower aspect ratio (AR), and they more likely to be located in PcoA or MCA, reported less history of CAD, more female and longer duration time of diabetes. Besides, patients in the rupture group had a more irregular shape and wide neck than the those in the non-rupture group. In addition, we also present the variable distribution results after PS matching in Table 1. We used PS 0.05 as the caliper value and matched at a ratio of 1:1 (see Supplementary Table 1 for matching methodological parameters). Finally, 64 pairs of patients were successfully matched. After matching, no statistically significant differences were detected in the distribution of these variables between patients with ruptured aneurysms and those without. This proves that the PS matching basically achieves its purpose. In addition, we also observed slight changes in the proportions of some categorical variables and values of continuous variables after matching, but the changes were all <10%.

### Results of Unadjusted and Adjusted Binary Logistic Regressions

In this study, we constructed two models to analyze the independent association of GHB with risk of IA rupture with univariate and multivariate binary logistic regression models. OR and 95% CI are listed in Table 2. In the unadjusted model, the model-based OR can be explained as the difference in per 1% change of GHB associated with differences in risk of IA rupture. For example, a 1.1 OR means that a difference in per 1% change in GHB is associated with increase of 10% in risk of IA rupture (1.1, 95% CI 0.94–1.29). In the fully adjusted model (all covariates presented in Table 1 were adjusted), GHB difference was not associated with IA rupture (1.07, 95% CI 0.84–1.35). For the purpose of sensitivity analysis, we performed PS matching and IPTW to verify our results (Table 2). Although there is a slight change in the range of ORs and CIs, the direction of the ORs has not changed, and the CI is roughly the same. We also converted GHB from continuous variable to categorical variable (quartile), the *P* for the trend of GHB with categorical variables in the fully adjusted model was consistent with the result when GHB was handled as continuous variable. Besides, we also found that the trend of the OR among different GHB groups was non-equidistant. This non-equidistant variation of OR values indicates the possibility of nonlinearity.

**Table 2:** Trend of model-based odds ratio (OR) in the unadjusted, fully adjusted, propensity score (PS)-matched, and inverse probability treatment weighting (IPTW) models.

**Exposure**	**Non-adjusted model**	**Fully-adjusted model**	**PS-matched model**	**IPTW model**
	**OR, 95%CI**	**OR, 95%CI**	**OR, 95%CI**	**OR, 95%CI**
GHB	1.10 (0.94, 1.29)	1.07 (0.84, 1.35)	1.02 (0.73, 1.15)	1.05 (0.96, 1.19)
**GHB (quartile)**
Q1 (1.88–5.96%)	Ref	Ref	Ref	Ref
Q2 (6.02–7.40%)	1.11 (0.51, 2.45)	1.06 (0.34, 3.24)	1.06 (0.35, 2.89)	1.29 (0.74, 2.20)
Q3 (7.44–8.59%)	1.58 (0.73, 3.42)	1.68 (0.54, 5.20)	1.09 (0.34, 3.36)	1.50 (0.87, 2.56)
Q4 (8.60–10.14%)	1.47 (0.68, 3.19)	1.65 (0.54, 5.00)	1.22 (0.24, 2.50)	1.20 (0.70, 2.06)
*P* for trend	0.227	0.277	0.779	0.436

*OR, odds ratio; CI, confidence interval; PS, propensity score; IPTW, inverse probability treatment weighting*.

*Non-adjusted model: no covariates were adjusted*.

*Fully adjusted model: we adjusted for all the covariates presented in Table 1*.

### Results of Nonlinearity of GHB and Risk of IA Rupture

**Figure 1:**
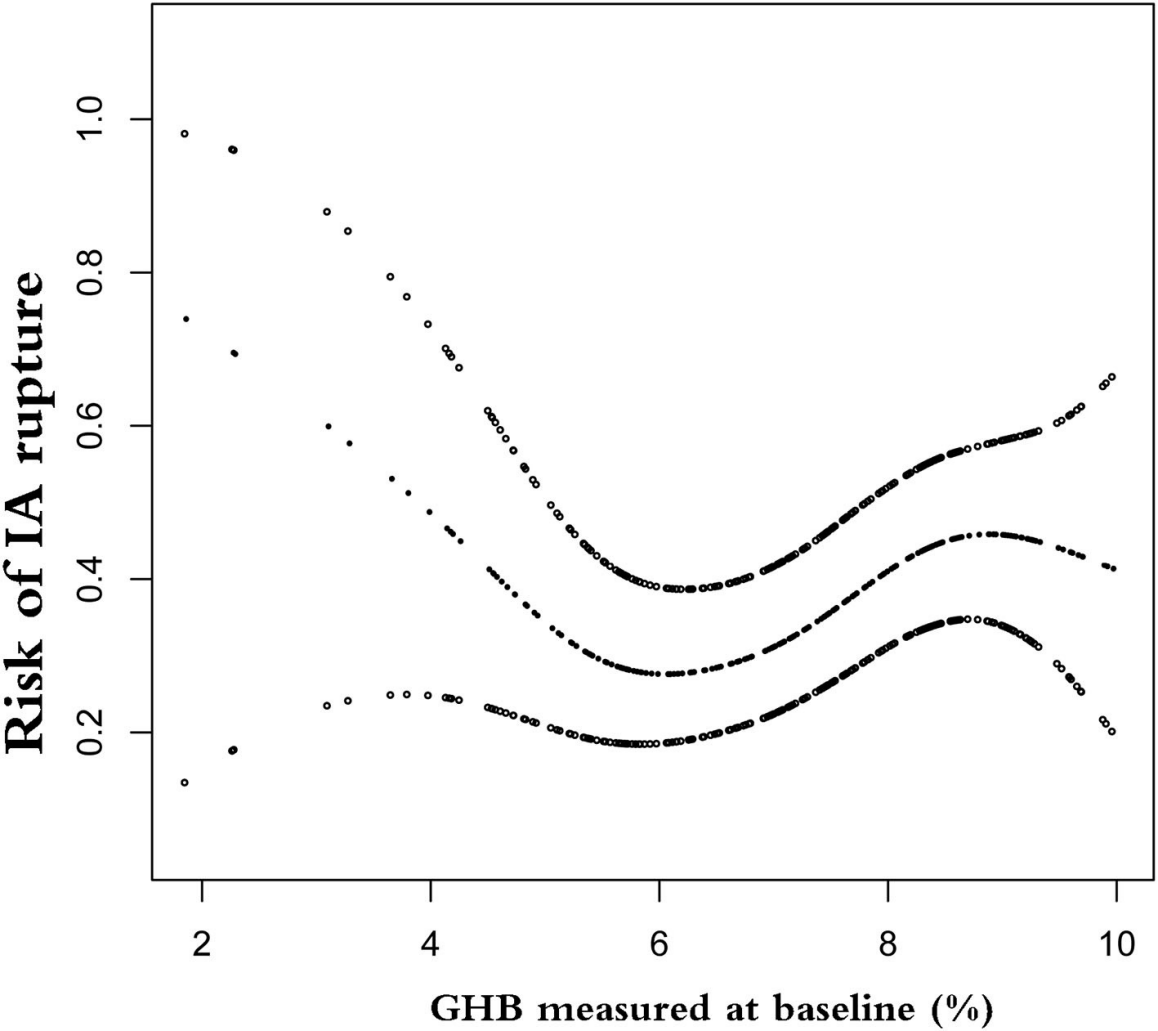
Nonlinear relationship between glycosylated hemoglobin (GHB) and risk of intracranial aneurysm (IA) rupture. The horizontal axis on the graph is the value of GHB, and the vertical axis is the risk of IA rupture.

In this study, we further explored the nonlinear relationship between GHB and rupture of IA (Figure 1). The smooth curve fitting indicated that the relationship between GHB and IA rupture was nonlinear and independent of demography, morphology, comorbidities, and lifestyle. Using two-piecewise binary logistic regression and recursive algorithms, we calculated the inflection points of GHB, which were 5.5 and 8.9%. In the range of 1.88–5.5%, GHB was negatively associated with risk of rupture (OR 0.38; 95% CI 0.16–0.9); in the range of 5.5–8.9%, per 1% elevated GHB was associated with 60% increase in risk of IA rupture (OR 1.6; 95% CI, 1.03–2.50); in the range of 8.9–10.1% of GHB, the association of GHB with IA rupture was not detected (OR 0.56; 95% CI 0.06–5.34) (Table 3).

**Table 3:** Addressing the nonlinearity of glycosylated hemoglobin with IA ruptured status.

	**OR, 95%CI**	***P*-values**
Fitting model using standard binary logistic regression model	1.07 (0.84, 1.35)	0.584
**Fitting model using two-piecewise binary logistic regression model**		
Inflection points calculated by recursive algorithm;	5.5, 8.9	
≤ 5.5%	0.38 (0.16, 0.90)	0.029
>5.5– ≤ 8.9%	1.60 (1.03, 2.50)	0.038
>8.9%	0.56 (0.06, 5.34)	0.615
*P* for log likely ratio test	0.012	

## Discussion

In summary, this analysis found a nonlinear correlation between IA rupture and GHB in Chinese patients with DM harboring single IA. Combining the trend of OR values in different GHB ranges and referring to the normal range of Chinese GHB (4–6%), our findings suggest that for Chinese patients with DM and single IA, good and stable glycemic control may be associated with reduced risk of IA rupture. Conversely, a high glycosylated hemoglobin ratio will significantly increase the risk of IA rupture.

The association between DM and IA rupture risk has been confusing. Lindgren et al. (6) reviewed eight studies investigating the correlation between DM and risk of IA rupture, with the size of the studies ranging from 254 to 1,596 patients. The pooled OR obtained from the fixed-effect model suggests that DM is a prospective factor in IA rupture. However, in the same study, they used their own data but found that DM was not associated with IA rupture. Other mechanism-driven studies have indicated that hyperglycemia is the main cause of vascular lesion. Besides, hyperglycemia also leads to vascular endothelial damage and dysfunction, and decrease in cerebral tight junction protein expression (1–5). Therefore, the negative link of DM to IA rupture is unexplained.

A large sample-size case-control study (*n* = 4,701) reported by Can et al. (7) explained this paradox. They suggest that unadjusted key confounders, unclear use of hypoglycemic drugs, imprecise diagnosis of diabetes, and lower IA rupture ratios have a bias on their results. In the study of Can et al. (7), they concluded that glucose-lowering agents are associated with decreased risk of aneurysmal subarachnoid hemorrhage, and that GHB levels were not significantly associated with rupture status. However, they did not explain why GHB is not associated with IA rupture, and why the use of hypoglycemic agents reduced the risk of IA rupture. In that study, Can et al. (7) evaluated hypoglycemic effect values calculated in the total population. However, those who do not have diabetes do not take hypoglycemic agents; In contrast, the association of GHB with aneurysm rupture is estimated in a population with diabetes only (4,062 cases of GHB were missing). Of particular importance is that they have not considered the nonlinear relationship between GHB and risk of IA rupture.

In our study, we restricted the study population to the DM population and assessed the association of GHB with IA rupture risk. Combining the Chinese GHB normal value reference range, we found that when GHB is within the approximate normal range, the risk of IA rupture decreases with increase in GHB. The results indicate that for diabetic patients with single IA, GHB is best controlled between 4 and 6 (not as low as possible). Conversely, in patients with poor glycemic control, GHB is positively associated with IA rupture risk, which indirectly confirms the results of Anil Can et al.; that is, use of hypoglycemic agents reduces the risk of IA rupture. However, this positive correlation has a saturation effect; that is, when GHB is >9, even if GHB increases again, the risk of IA rupture does not increase further.

This research has several strengths of note. First, compared to most previous studies that only adjust morphological parameters or demographic characteristics, the adjustment strategy of this study is more complete and sufficient. Second, to our best knowledge, it is the first to address nonlinearity and test interaction on the association of GHB with IA rupture. Nonlinearity addressing makes our results have more clinical value. Third, the sensitivity analysis ensures the robustness of our findings. PS matching and IPTW are a good proof that our results regarding the linear association between GHB and IA rupture risk are robust.

Indeed, interpretation of the findings of this study should be made with caution. Several limitations are also noteworthy. First, conclusions can be generalized to patients with single IA only, and correlation of GHB with ruptured IA may be different in multiple IAs. Second, limited to actual clinical conditions, this study was only designed as a cross-sectional one, so the causal association between GHB and IA rupture could not be confirmed. However, GHB reflects long-term blood glucose control and is better than fasting blood glucose (high intra-individual variability). We can, therefore, think of GHB as one of the factors that influence the risk of IA rupture. Third, although we have adjusted for measurable confounders, as in all observational studies, there still might have been uncontrolled confounding due to unmeasured differences between IA with and IA without rupture. Although PSM and IPTW can weaken the impact of non-measured confounding factors on outcomes (21), they cannot be completely avoided.

## Conclusion

For patients with single IA and DM, good glycemic control can effectively reduce the risk of IA rupture, and the results are independent of known risk factors such as demographics, morphology, lifestyle, and comorbidities.

## Data Availability Statement

The raw data supporting the conclusions of this article will be made available by the authors, without undue reservation.

## Ethics Statement

The studies involving human participants were reviewed and approved by Zhujiang Hospital of Southern Medical College. The Ethics Committee waived the requirement of written informed consent for participation.

## Author Contributions

X-FL completed the manuscript and participated in the study design. X-TW participated in data collection and data analysis. S-XS participated in research design and paper review. XZ provided research funding and participated in study design and manuscript writing. Y-MB participated in data collection. C-ZD participated in the study design and review of the paper. All authors contributed to the article and approved the submitted version.

## Funding

This study was supported by the National Natural Science Foundation Project (Grant No: 81974177) and the Initiation Plan Project of Clinical Research of Southern Medical University (Grant No: QD2018N022).

## Conflict of Interest

The authors declare that the research was conducted in the absence of any commercial or financial relationships that could be construed as a potential conflict of interest.

## Publisher's Note

All claims expressed in this article are solely those of the authors and do not necessarily represent those of their affiliated organizations, or those of the publisher, the editors and the reviewers. Any product that may be evaluated in this article, or claim that may be made by its manufacturer, is not guaranteed or endorsed by the publisher.

## References

[1] Ding C Toll V Ouyang B Chen M. Younger age of menopause in women with cerebral aneurysms. J Neurointerv Surg. (2013) 5:327–31. 10.1136/neurintsurg-2012-010364 22700728

[2] Li W Prakash R Prakash R Kelly-Cobbs AI Ogbi S Kozak A. Adaptive cerebral neovascularization in a model of type 2 diabetes: relevance to focal cerebral ischemia. Diabetes. (2010) 59:228–35. 10.2337/db09-0902 19808897 PMC2797926

[3] Ye X Chopp M Cui X Zacharek A Cui Y Yan T. Niaspan enhances vascular remodeling after stroke in type 1 diabetic rats. Exp Neurol. (2011) 232:299–308. 10.1016/j.expneurol.2011.09.022 21963653 PMC3265018

[4] Tamura T Jamous M A Kitazato K T Yagi K Tada Y Uno M. Endothelial damage due to impaired nitric oxide bioavailability triggers cerebral aneurysm formation in female rats. J Hypertens. (2009) 27:1284–92. 10.1097/HJH.0b013e328329d1a7 19307983

[5] Tada Y Yagi K Kitazato K T Tamura T Kinouchi T Shimada K. Reduction of endothelial tight junction proteins is related to cerebral aneurysm formation in rats. J Hypertens. (2010) 28:1883–91. 10.1097/HJH;0b013e32833c2273 20577123

[6] Lindgren A E Kurki M I Riihinen A Koivisto T Ronkainen A Rinne J. Type 2 diabetes and risk of rupture of saccular intracranial aneurysm in eastern Finland. Diabetes Care. (2013) 36:2020–6. 10.2337/dc12-1048 23536581 PMC3687302

[7] Can A Castro V M Yu S Dligach D Finan S Gainer VS. Antihyperglycemic agents are inversely associated with intracranial aneurysm rupture. Stroke. (2018) 49:34–9. 10.1161/STROKEAHA.117.019249 29203688

[8] Peters A Laffel L American Diabetes Association Transitions Working Group. Diabetes care for emerging adults: recommendations for transition from pediatric to adult diabetes care systems: a position statement of the American Diabetes Association, with representation by the American College of Osteopathic Family Physicians, the American Academy of Pediatrics, the American Association of Clinical Endocrinologists, the American Osteopathic Association, the Centers for Disease Control and Prevention, Children with Diabetes, The Endocrine Society, the International Society for Pediatric and Adolescent Diabetes, Juvenile Diabetes Research Foundation International, the National Diabetes Education Program, and the Pediatric Endocrine Society (formerly Lawson Wilkins Pediatric Endocrine Society). Diabetes Care. (2011) 34:2477–85. 10.2337/dc11-1723 22025785 PMC3198284

[9] Zhang X Yao Z Q Karuna T Duan CZ Wang XM Li XF. Cerebral microbleeds could be independently associated with intracranial aneurysm rupture: a cross-sectional population-based study. World Neurosurg. (2018) 115:e218–25. 10.1016/j.wneu.2018.04.018 29654957

[10] Zhang X Long XA Luo B Karuna T Duan CZ. Factors responsible for poor outcome after intraprocedural rerupture of ruptured intracranial aneurysms: identification of risk factors, prevention and management on 18 cases. Eur J Radiol. (2012) 81:e77–85. 10.1016/j.ejrad.2011.02.015 21353424

[11] Can A Castro V M Dligach D Finan S Yu S Gainer V. Lipid-lowering agents and high HDL (high-density lipoprotein) are inversely associated with intracranial aneurysm rupture. Stroke. (2018) 49:1148–54. 10.1161/STROKEAHA.117.019972 29622625 PMC5915939

[12] You SH Kong DS Kim JS Jeon P Kim KH Roh HK. Characteristic features of unruptured intracranial aneurysms: predictive risk factors for aneurysm rupture. J Neurol Neurosurg Psychiatry. (2010) 81:479–84. 10.1136/jnnp.2008.169573 19726404

[13] Backes D Vergouwen MD Velthuis BK van der Schaaf IC Bor AS Algra A. Difference in aneurysm characteristics between ruptured and unruptured aneurysms in patients with multiple intracranial aneurysms. Stroke. (2014) 45:1299–303. 10.1161/STROKEAHA.113.004421 24652309

[14] Björkman J Frösen J Tähtinen O Backes D Huttunen T Harju J. Irregular shape identifies ruptured intracranial aneurysm in subarachnoid hemorrhage patients with multiple aneurysms. Stroke. (2017) 48:1986–9. 10.1161/STROKEAHA.117.017147 28468927

[15] Millan R D Dempere-Marco L Pozo J M Cebral J R Frangi A F. Morphological characterization of intracranial aneurysms using 3-D moment invariants. IEEE Trans Med Imaging. (2007) 26:1270–82. 10.1109/TMI.2007.901008 17896598

[16] Elsharkawy A Lehečka M Niemelä M Kivelev J Billon-Grand R Lehto H. Anatomic risk factors for middle cerebral artery aneurysm rupture: computed tomography angiography study of 1009 consecutive patients. Neurosurgery. (2013) 73:825–37. 10.1227/NEU.0000000000000116 24141397

[17] Mocco J Brown RD Jr Torner JC Capuano AW Fargen KM Raghavan ML. Aneurysm morphology and prediction of rupture: an international study of unruptured intracranial aneurysms analysis. Neurosurgery. (2018) 82:491–6. 10.1093/neuros/nyx226 28605486 PMC6256940

[18] Ujiie H Tachibana H Hiramatsu O HazelA L Matsumoto T Ogasawara Y. Effects of size and shape (aspect ratio) on the hemodynamics of saccular aneurysms: a possible index for surgical treatment of intracranial aneurysms. Neurosurgery. (1999) 45:119–29. 10.1227/00006123-199907000-00028 10414574

[19] Matsukawa H Kamiyama H Kinoshita Y Saito N Hatano Y Miyazaki T. Morphological parameters as factors of 12-month neurological worsening in surgical treatment of patients with unruptured saccular intracranial aneurysms: importance of size ratio. J Neurosurg. (2018) 131:852–8. 10.3171/2018.4.JNS173221 30239320

[20] Yao XI Wang X Speicher PJ Hwang ES Cheng P Harpole DH. Reporting and guidelines in propensity score analysis: a systematic review of cancer and cancer surgical studies. JNCI. (2017) 109:djw323. 10.1093/jnci/djw323 28376195 PMC6059208

[21] Borah BJ Moriarty JP Crown WH Doshi JA. Applications of propensity score methods in observational comparative effectiveness and safety research: where have we come and where should we go? J Comp Eff Res. (2014) 3:63–78. 10.2217/cer.13.89 24266593

[22] Austin P C. An introduction to propensity score methods for reducing the effects of confounding in observational studies. Multivariate Behav Res. (2011) 46:399–424. 10.1080/00273171.2011.568786 21818162 PMC3144483

